# Gastrointestinal Hemorrhage due to Large B-Cell Lymphoma Treated With Hemospray

**DOI:** 10.7759/cureus.8528

**Published:** 2020-06-09

**Authors:** Gurbir Sehmbey, Dimas Kosa, Indu Srinivasan, Keng-Yu Chuang

**Affiliations:** 1 Internal Medicine, Banner University Medical Center, Phoenix, USA; 2 Internal Medicine, University of Arizona College of Medicine - Phoenix, Phoenix, USA; 3 Internal Medicine, Valleywise Health Medical Center, Phoenix, USA; 4 Gastroenterology, Valleywise Health Medical Center, Phoenix, USA; 5 Internal Medicine/Gastroenterology, Valleywise Health Medical Center, Phoenix, USA; 6 Internal Medicine/Gastroenterology, Creighton University School of Medicine-Phoenix Program, Phoenix, USA

**Keywords:** gi bleeding, tumor, bowel perforation, over the scope clips, endoscopy

## Abstract

Gastrointestinal bleeding (GIB) is a common cause of hospitalization and is associated with significant morbidity and mortality. The most frequent causes of nonvariceal upper GIB are peptic ulcers, mucosal erosions, Mallory-Weiss tears, and malignancy. Current endoscopic hemostatic methods, including injections, thermal and mechanical modalities, have a 5%-10% chance of recurrent bleeding. Hemospray (Cook Medical, Winston-Salem, NC, USA) is a recently approved modality and can help treat tumor-related GIB. We present a case of a patient with diffuse large B-cell gastric lymphoma who presented with tumor-related GIB. His clinical course was complicated by gastric perforation and active bleeding which was treated with Hemospray and over-the-scope clips (OTSC, Ovesco, Tübingen, Germany).

## Introduction

Gastrointestinal bleeding (GIB) is a potentially life-threatening condition and is one of the main indications of emergent endoscopy. It has an incidence of 150 patients per 100,000 population and is associated with significant morbidity and mortality [1]. Common causes of GIB include ulcers, esophageal varices, vascular malformations, and malignancy [1]. Various therapy techniques exist to achieve hemostasis; however, current conventional endoscopic treatments provide unsatisfactory outcomes in upper GIB due to tumor [2]. Hemospray TC-325 (Cook Medical, Winston-Salem, NC, USA) is a powder agent, which is a promising therapy for hemostasis in upper GIB from a tumor [3].

## Case presentation

**Figure 1:**
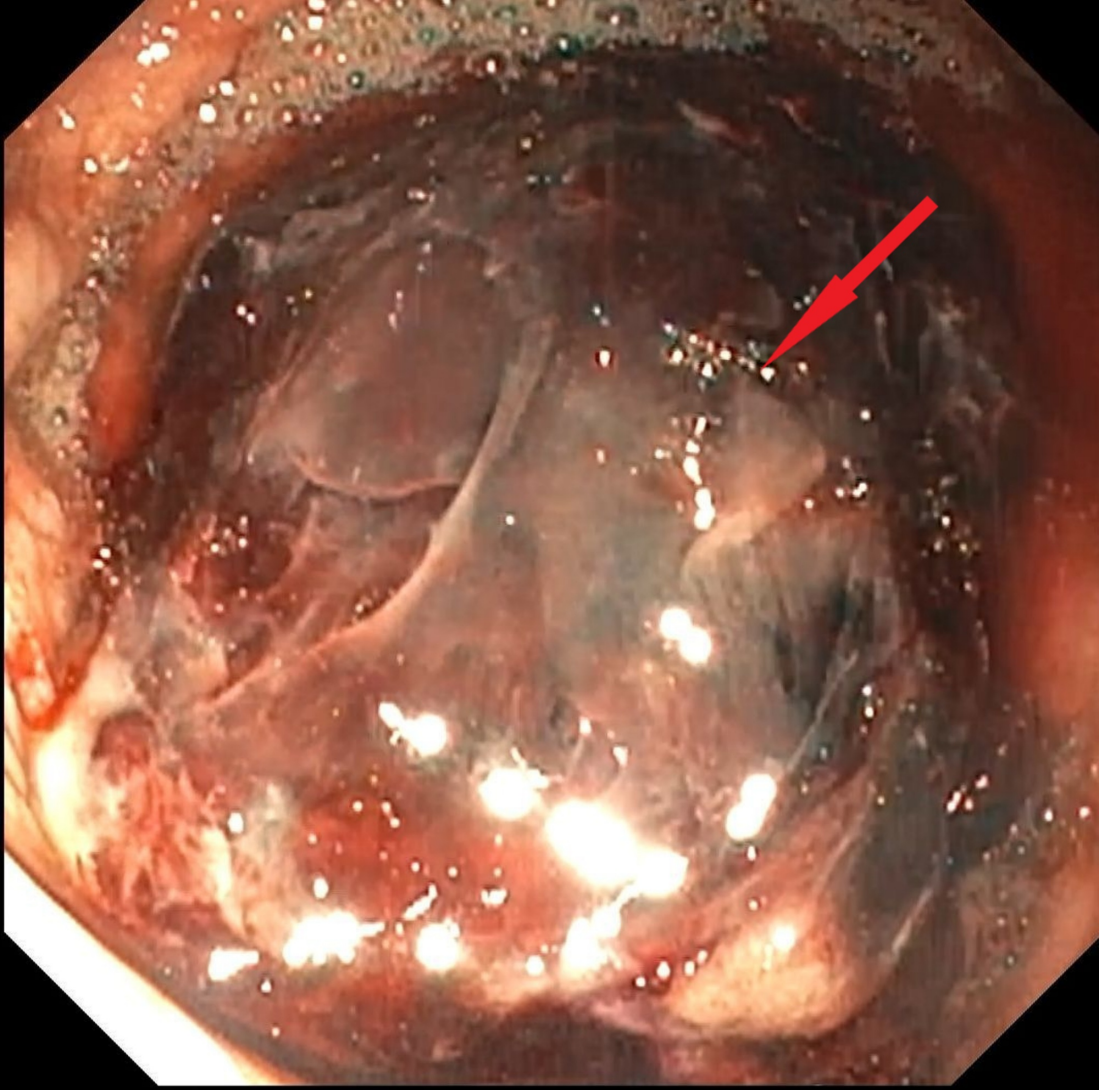
Esophagogastroduodenoscopy showing a large blood clot attached to a giant ulcer in the great curvature of the fundus without active bleeding.

A 57-year-old male with a history of heroin abuse, chronic hepatitis C, and advanced diffuse large B-cell gastric lymphoma with a contained perforation on chemotherapy presented to the hospital with dizziness, hematemesis, and abdominal pain. On arrival, the patient was noted to be hypotensive with a blood pressure of 79/50 mmHg and tachycardic with a heart rate of 139 bpm. On physical exam, the patient had epigastric tenderness, dull bowel sounds, and splenomegaly. Labs revealed hemoglobin (Hgb) 8.2 g/dL, international normalized ratio (INR) 1.6, and lactic acid 3.2 mg/dL. CT of the abdomen revealed a gastric mass in the greater curvature of the stomach with a 3.1 cm perforation along with free fluid and air in the abdomen. The patient was admitted to the intensive care unit (ICU), and resuscitation was initiated with intravenous pantoprazole and red blood cell transfusion. Esophagogastroduodenoscopy (EGD) was performed revealing a large blood clot attached to a giant ulcer in the great curvature of the fundus (Figure 1) without active bleeding. No endoscopic intervention was performed, and oral sucralfate was added to therapy. The patient’s Hgb stabilized after blood transfusions, and the patient was transferred to the floor.

Two days later, the patient experienced massive hematemesis, abdominal pain, and decreased mental status. Vitals revealed hemodynamic instability with a blood pressure of 65/50 mmHg and heart rate 140 bpm. Labs revealed Hgb of 6.5 g/dL. The patient was intubated for airway protection and emergent blood transfusion was initiated along with inotrope therapy for the treatment of hemorrhagic shock. Given active bleeding, a visceral arteriogram was performed, revealing active bleeding from the splenic artery which was treated with embolization and coiling. After the procedure, the patient’s Hgb was monitored in the ICU.

**Figure 2:**
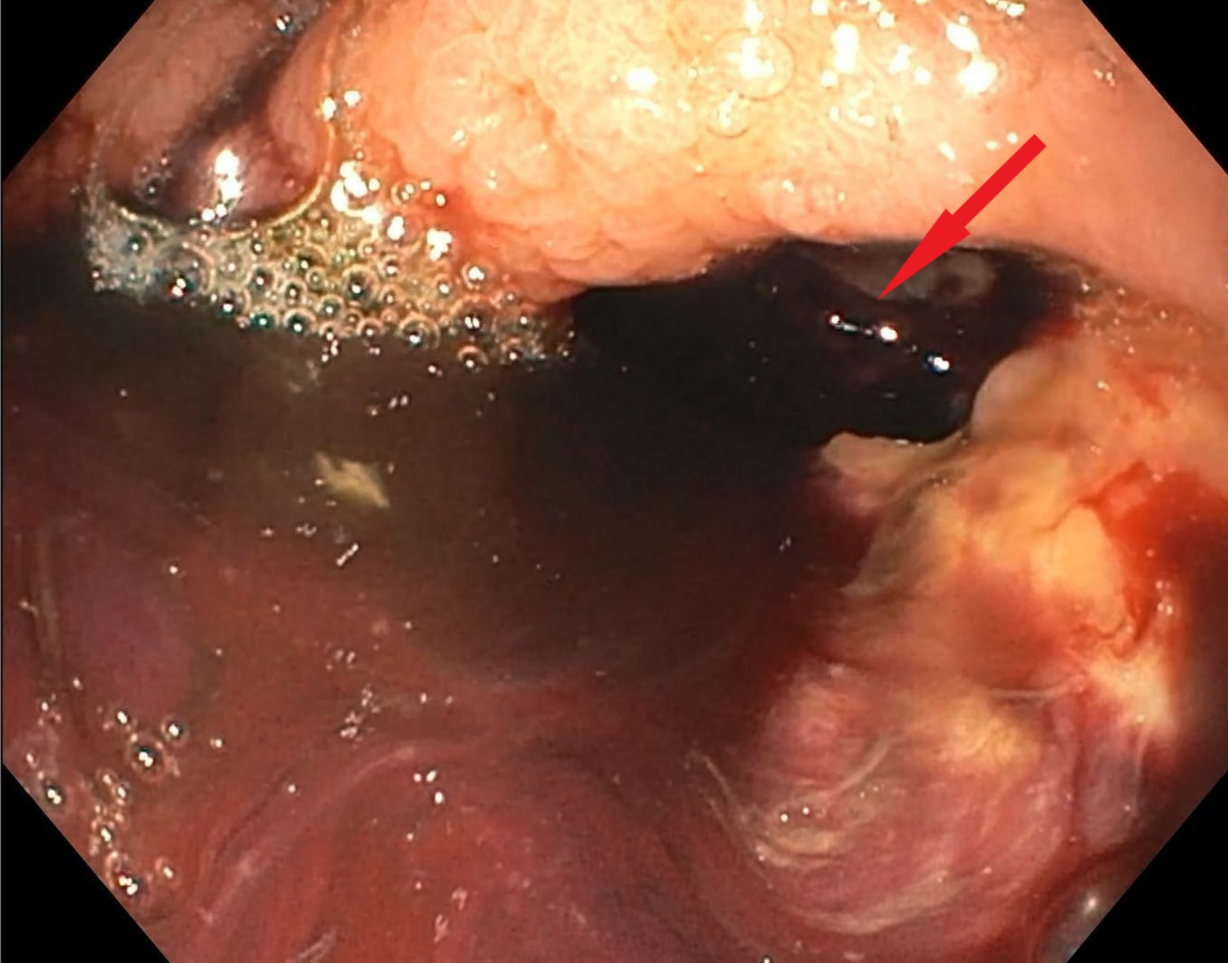
Repeat esophagogastroduodenoscopy showing a large crater ulcer in the fundus/greater curvature of the gastric body and a large perforation with evidence of recent bleeding.

Over the next day, the patient’s Hgb continued to decrease, requiring more blood transfusions. A repeat EGD showed a large crater ulcer in the fundus/greater curvature of the gastric body and a large perforation with evidence of recent bleeding (Figure 2).

**Figure 3:**
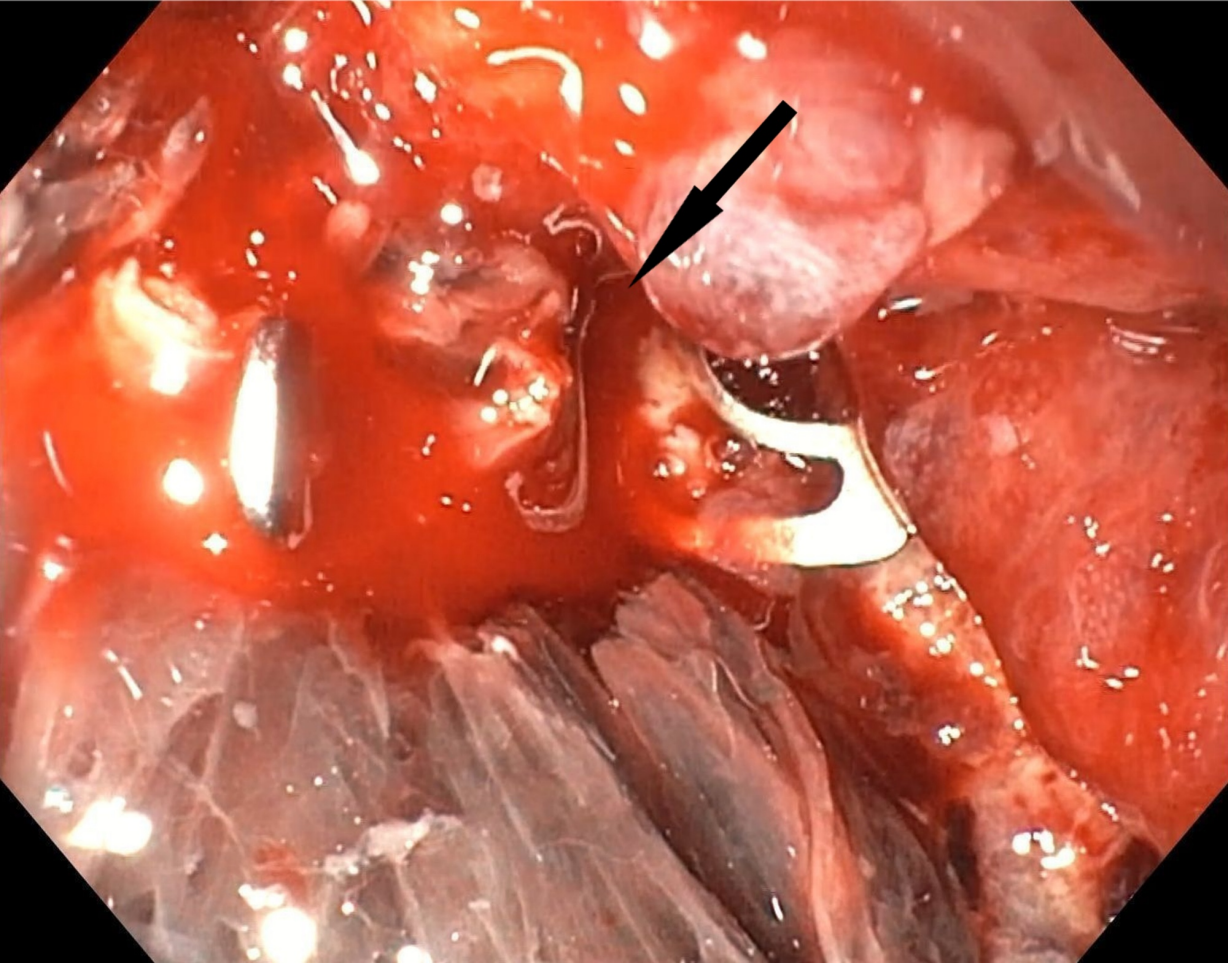
Large gastric perforation closed using two OTSCs (Ovesco, Tübingen, Germany). OTSC, over the scope clip.

Initial attempts to approximate the edges of perforation using a dual grasper were unsuccessful due to the gastric mucosa being friable. Therefore, the suction method was used to approximate the edges, and two OTSCs (Ovesco, Tübingen, Germany) were used to close the perforation (Figure 3). 

**Figure 4:**
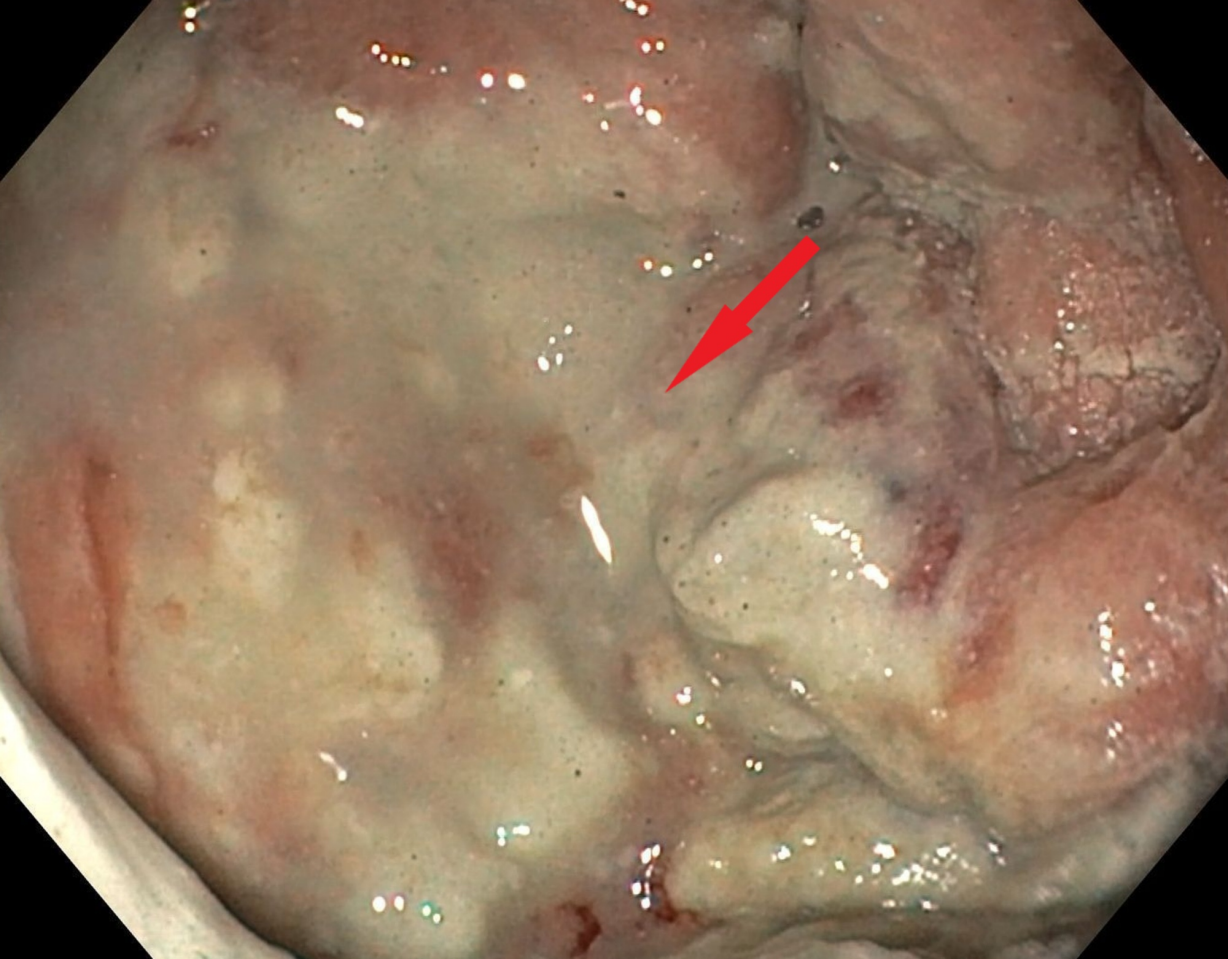
Hemospray (Cook Medical, Winston-Salem, NC, USA) deployed over the entire ulcer achieving hemostasis.

At this point, Hemospray was deployed achieving hemostasis of the entire ulcer (Figure 4). 

After the EGD, CT abdomen revealed a decreased amount of intraperitoneal free air without evidence of new perforation and no active bleeding. Over the next 24 hours, the patient’s Hgb stabilized, and no further blood products were transfused. Repeat EGD two days later showed persistent contained perforation without any active bleeding. The patient was discharged home and was cleared to resume chemotherapy. A follow-up EGD was performed after two months of chemotherapy, which revealed a marked decrease in the size of the ulcer and nearly resolved contained perforation [4]. 

## Discussion

Tumor-related bleeding accounts for 2%-4% of nonvariceal upper GIB [5]. Tumor-related GIB can occur from both primary and metastatic tumors in the GI tract [6]. Bleeding due to a tumor can be diffuse and extensive as it is commonly induced by tumor necrosis [6]. Tumor-related bleeding becomes a challenging problem to control with conventional endoscopic hemostatic techniques, such as argon plasma coagulation, electrocautery, and mechanical hemostasis [7]. Conventional therapies are associated with high rates of re-bleeding in tumor-related GIB [7]. Hemospray is a FDA-approved modality that can help control tumor-related GIB by providing temporary hemostasis while allowing time for a definitive treatment, such as surgery, chemotherapy, or radiation [8].

Hemospray is an inorganic powder, which when in contact with blood, absorbs water and adhesively forms a barrier over the bleeding site. Hemospray is not absorbed or metabolized by mucosal tissue [8]. The powder forms a barrier due to the accumulation of clotting factors that protects the bleeding point from the acid allowing healing to occur [9]. No local or systemic side effects of this therapy have been reported [10-12].

Hemospray, unlike traditional therapies, is a nonthermal, nontraumatic, and noncontact modality that does not require precise targeting of other endoscopic devices [13]. It is delivered by a carbon dioxide delivery system through a catheter inserted into the working channel of the endoscope. Hemospray has a quick onset of action. It can be applied in short one- to two-second bursts until the bleeding site is completely covered with powder and no active bleeding is visualized. Hemospray is not absorbed by the body and usually passes through the lower GI tract within 72 hours [13]. Overall, the spray is 95% effective to achieve hemostasis in cases with upper and lower GI bleed [10,14]. Hemospray has been found to be safe with a seven-day rebleeding risk of 20% and bowel perforation risk of 0.9% [11,14]. Therefore, Hemospray can be a promising therapy for initial hemostasis in tumor-related bleeding.

## Conclusions

GIB due to a tumor is difficult to control with conventional endoscopic techniques; therefore, there is much need for reliable bridging therapy. Hemospray can be used for initial hemostasis in high-risk cases, such as upper GIB from a tumor, as a temporary measure allowing sufficient time toward more definitive therapy. Hemospray is a safe and promising therapy that can be used as first-line treatment and also as salvage therapy with good short-term effectiveness.
